# Efficacy and safety of Levamisole treatment in clinical presentations of non-hospitalized patients with COVID-19: a double-blind, randomized, controlled trial

**DOI:** 10.1186/s12879-021-05983-2

**Published:** 2021-03-24

**Authors:** Amirreza Roostaei Firozabad, Zohreh Akhoundi Meybodi, Seyed Ruhollah Mousavinasab, Adeleh Sahebnasagh, Mohsen Gholinataj Jelodar, Iman Karimzadeh, Solomon Habtemariam, Fatemeh Saghafi

**Affiliations:** 1 Pharmaceutical Sciences Research Center, Student Research Committee, School of Pharmacy, Shahid Sadoughi University of Medical Sciences and Health Services, Yazd, Iran; 2 Infectious disease research center, Shahid Sadoughi hospital, Shahid Sadoughi University of Medical Sciences, Yazd, Iran; 3 Resident of Clinical Pharmacy, Department of Clinical Pharmacy, School of Pharmacy, Shiraz University of Medical Sciences, Shiraz, Iran; 4 Clinical Research Center, Department of Internal Medicine, School of Medicine, North Khorasan University of Medical Sciences, Bojnurd, Iran; 5 Department of Internal Medicine, School of Medicine, Shahid Sadoughi University of Medical Sciences, Yazd, Iran; 6 Department of Clinical Pharmacy, School of Pharmacy, Shiraz University of Medical Sciences, Shiraz, Iran; 7 Pharmacognosy Research Laboratories and Herbal Analysis Services UK, University of Greenwich, Central Avenue, Chatham-Maritime, Kent, ME4 4TB UK; 8 Department of Clinical Pharmacy, Faculty of Pharmacy and Pharmaceutical Sciences Research Center, Shahid Sadoughi University of Medical Sciences, Yazd, Iran

**Keywords:** Levamisole, Hydroxychloroquine, COVID-19, SARS-CoV2, Clinical status

## Abstract

**Background:**

Levamisole has shown clinical benefits in the management of COVID-19 via its immunomodulatory effect. However, the exact role of Levamisole effect in clinical status of COVID-19 patients is unknown. We aimed to evaluate the efficacy of Levamisole on clinical status of patients with COVID-19 during their course of the disease.

**Methods:**

This prospective, double-blind, randomized controlled clinical trial was performed in adult patients with mild to moderate COVID-19 (room-air oxygen saturation > 94%) from late April 2020 to mid-August 2020. Patients were randomly assigned to receive a 3-day course of Levamisole or placebo in combination with routine standard of care.

**Results:**

With 25 patients in each arm, 50 patients with COVID-19 were enrolled in the study. Most of the study participants were men (60%). On days 3 and 14, patients in Levamisole group had significantly better cough status distribution when compared to the placebo group (*P*-value = 0.034 and 0.005, respectively). Moreover, there was significant differences between the two groups in dyspnea at follow-up intervals of 7 (*P*-value = 0.015) and 14 (*P*-value = 0.010) days after receiving the interventions. However, no significant difference in fever status was observed on days 1, 3, 7, and 14 in both groups (*P*-value > 0.05).

**Conclusion:**

The results of the current study suggest that Levamisole may improve most of clinical status of patients with COVID-19. The patients receiving Levamisole had significantly better chance of clinical status including cough and dyspnea on day 14 when compared to the placebo. However, the effect-size of this finding has uncertain clinical importance.

**Trial registration:**

The trial was registered as IRCT20190810044500N7 (19/09/2020).

## Background

In late December 2019, a novel human coronavirus, Severe Acute Respiratory Syndrome Coronavirus 2 (SARS-CoV-2 or COVID-19), was identified as the cause of a series of pneumonia cases in Wuhan, Hubei Province in China [1–3]. The outbreak has rapidly spread, resulting in an epidemic throughout China, as well as other countries around the world. On March 12th 2020, the World Health Organization (WHO) announced the outbreak of COVID-19 as a pandemic [4]. Predominant symptoms at the initiation of the disease include the following: fever, cough, myalgia, chills, dyspnea and pneumonia. Less common Symptoms are sputum, headache, hemoptysis and diarrhea [5].

Since the structure of the SARS-CoV and SARS-CoV-2 was similar, the biochemical interactions and the pathogenesis were also expected to be similar [6]. However, the pathogenic mechanism may proceed differently in the novel SARS-CoV-2 and the intermediate host of SARS-CoV2 is yet to be identified. In order to enter into the host cell, the virus needs to bind to the angiotensin-converting enzyme 2 (ACE-2) receptors in the type II pneumocytes in the lungs and form a viral endosome through a clathrin-mediated endocytosis [7–9]. When internalized, the fusion of virus with lysosomes occurs under low endosomal and lysosomal pH [10]. The binding of the SARS-CoV-2 to the ACE-2 receptors triggers an inflammation cascade in the lower respiratory tract and causes a systemic inflammatory state. Infection of human cells by SARS-CoV-2 results in inflammatory cascade by virus-infected antigen-presenting cells (APCs). APCs present the outsider antigen to CD4 + -T-helper (Th1) cells, and release interleukin-12 which further stimulate B-cells to produce antigen-specific antibodies [11, 12]. Currently, few approved prophylactic or therapeutic medications are available for COVID-19 diseases. Some therapeutic agents are used off-label, alone or in combination [13].

Levamisole is a synthetic low-molecular weight agent which belongs to the anti-helminthic class of medications. Levamisole can enhance cellular immunity depending on the dosage and timing of the administration [14, 15]. In in-vitro models, it has been reported that the combination of Levamisole and ascorbic acid can reverse the depressed helper/inducer subpopulation of lymphocyte in measles virus. The abnormality in lymphocytes number could be reproduced in Levamisole treated lymphocytes in-vitro [15, 16].

Through improving the cellular immunity, Levamisole showed efficacy in various autoimmune and inflammatory diseases [17]. Interestingly, this anti-helminthic medication is able to phagocytose the immune cells to the normal level via its immune regulatory properties. Furthermore, Levamisole improved the function of human interferon and modulates T-cell immunity. The efficacy of this drug has been further documented in the management of some malignancies and autoimmune diseases such as rheumatoid arthritis and systemic lupus erythematosus, vitiligo, and viral warts [18]. Levamisole inserts its promising effects on cellular and humoral immunity through regulating the circulating pro-inflammatory cytokines such as interleukins (IL) 6 and 8. Levamisole has also been used in patients with recurrent aphthous ulcers and it is believed to normalize the CD4 + / CD8 + cell ratio and elevates the serum levels of IgA and IgM.

Therefore, considering the pathophysiology of COVID-19 and the immunomodulatory properties of Levamisole, we conducted this double-blind, randomized controlled trial to evaluate the efficacy and safety of Levamisole when compared to the routine standard of care in non-hospitalized patients with mild to moderate COVID-19.

## Methods

This prospective, double-blind, randomized controlled clinical trial was performed in adult patients aged between 18 to 60 year old with mild to moderate COVID-19 (room-air oxygen saturation > 94%) who referred to the infection clinic of Shahid Sadoughi Hospital, Yazd, Iran. The diagnosis of COVID-19 was based on the results of polymerase chain reaction (PCR) (using the real-time PCR method with Pishtazteb kit, Pishtazteb company, Iran) and radiological manifestations of COVID-19 chest computed tomography (CT) scan during a 4 month period from late April 2020 to mid-August 2020. Patients were randomly assigned into two arms of the study by permutation block method: 25 patients in the control group and 25 patients in the intervention group. This study was approved by the Ethics Committee of Yazd University of Medical Sciences (Ethics ID: IR.SSU.REC.1399.063).

### Exclusion and inclusion criteria

Patients were included in the study if they met the following criteria: aged between 18 to 60 years old, diagnosis of COVID- 19 in the previous 24 h based on the clinical signs and symptoms and radiographical manifestations in lung CT scan, had not taken Levamisole during the previous 5 days (according to the 16-h half-life of the drug). In female patients, the eligible candidates were not being pregnant or planned pregnancy at least 30 days after the end of the study. The enrolled patients were also candidate for outpatient care and were informed not to take medications outside the study protocol. Patients were not included in this trial if they had the following criteria: hospitalization, hemodynamic instability, history of cirrhosis, hepatitis and severe liver diseases, severe renal failure (estimated glomerular filtration rate less than 30 mL/min), taking Levamisole for other indications (e.g., parasitic infections), history of allergic reaction or known allergy to Levamisole, history of receiving chemotherapy for cancer, Pregnant or lactating women.

### Intervention

Patients were randomly assigned into the following arms of the study by permutation block method: Levamisole with routine standard of care or placebo with routine standard of care. Routine standard of care consisted of hydroxychloroquine 200 mg twice daily for 5 days with acetaminophen 500 mg tablets to manage fever and diphenhydramine syrup 10 cc every 8 h for the management of cough. Patients in the intervention group took Levamisole 50 mg orally (manufactured by Rouzdarou) three times a day for 3 days in addition to the routine stands of care. Patients in the control group received placebo (placebo tablets were prepared in the Pharmaceutics Laboratory of Shahid Sadoughi School of Pharmacy, Yazd) in addition to the routine standard of care.

### Measurements

Demographic characteristics and the initial symptoms of the patients were recorded in the prepared questionnaire at baseline. Warning signs were explained to the patients for immediate referral and a pamphlet containing the contents was handed to them. Patients were contacted for follow-up visit in the clinic on the third and seventh days and were questioned about their clinical symptoms provided in the questionnaire. They were also asked about the use of acetaminophen for the management of fever, the extent of dyspnea and cough and their correlation with the level of activity, and the need for follow-up visit or hospitalization.

### Statistical analysis

The quantitative and qualitative variables were reported as mean ± standard deviation and number (frequency), respectively. The normally and non-normally distributed quantitative variables were compared between groups by using the independent Sample t-test and Mann-Whitney, respectively. Moreover, the qualitative variables were compared between two groups by using the Chi-Square test. We applied Fisher Exact test when the data sparsity was observed. All the statistical analysis was conducted by SPSS software version 20 and differences with a value of *P*-value < 0.05 were considered significant.

## Results

### Patients’ characteristics

Between April 12, 2020, and August 2, 2020, 59 patients with mild to moderate COVID-19 (room-air oxygen saturation > 94%) were evaluated for eligibility. From these patients, 50 were considered eligible. 25 patients were assigned to receive Levamisole along with the routine standard of care and 25 patients to receive placebo in addition to the routine care. The mean ± SD age of the patients in this study was 36.68 ± 13.33 years, and 60% of the patients were male. There were no significant differences in demographic characteristics between groups. Patients were balanced in baseline demographics characteristics in the two groups (Table 1).

**Table 1:** Patients Demographic Data

Characteristics	Placebo/Routine Care	Levamisole/ Routine Care
**Sex, No. (%)**		
Male	13 (52.0)	17 (68.0%)
Female	12 (48.0)	8 (32.0%)
**Age, median (IQR), y**		
	32.0 (15)	30.0 (14)
**Weight, median (IQR), kg**		
	70.0 (18)	74.0 (18)
**Marital status, No. (%)**		
Single	6 (24.0)	6 (24.0)
Married	19 (76.0)	19 (76.0)
**Smoking, No. (%)**		
Yes	2 (8.0)	4 (16.0)
No	23 (92.0)	21 (84.0)
**Mean of room-air O2 Sats, %**		
	96.50	96.33
**Lung CT-Scan involvement, No. (%)**		
Positive	22 (88.0)	22 (88.0)
Negative	0 (0.0)	2 (8.0)
None	3 (12.0)	1 (4.0)
**Lung CT-Scan involvement Score, %**		
	2.50	2.75
**PCR, No. (%)**		
Positive	24 (96.0)	22 (88.0)
Negative	1 (4.0)	3 (12.0)
**Coexisting conditions, No. (%)**		
Cardiovascular disease	1 (4.0)	0 (0.0)
Hypertension	2 (8.0)	1 (4.0)
Diabetes	3 (12.0)	1 (4.0)
Asthma	0 (0.0)	2 (8.0)
Hypothyroidism	1 (4.0)	0 (0.0)

*No* Number, *y* year, *kg* kilogram, *O2 Sats* oxygen saturation, *CT-Scan* Computed Tomography Scan, *CT involvement Score* Scoring the severity by the percentages of each of the five lobes that is involved: 1 for < 5%, 2 for 5–25%, 3 for 26–49%, 4 for 50–75%, and > 75% involvement. The total score is the sum of the individual lobar scores [19], *PCR* polymerase chain reaction

**Fig. 1:**
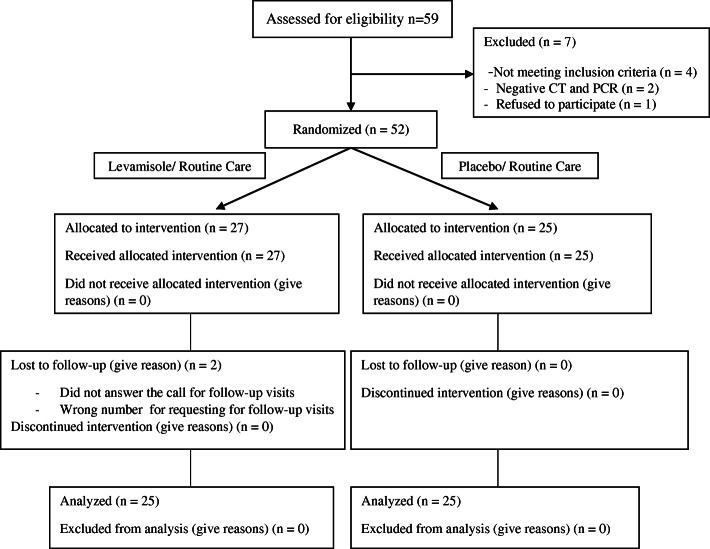
CONSORT flow diagram

From 59 patients who consented and were assessed for eligibility, 52 underwent randomization and initiate the study: 27 were assigned to the Levamisole/routine care group and 25 patients continued routine care with placebo (Fig. 1). Two patients withdrew the study due to wrong number and not answer the phone for follow-up visits, and 50 patients completed the trial. A CONSORT flow diagram of the study is presented in Fig. 1.

### Clinical improvement during Levamisole treatment

On days 3 and 14, patients randomized to the Levamisole/routine care group had significantly better cough status when compared with those randomized to placebo/routine care (*P*-value = 0.034 and 0.005, respectively). The differences in fever status on days 1, 3, 7, and 14 between the Levamisole/routine care and placebo/routine care groups were not statistically significant (*P* >.

0.05). There were significant differences between two groups in dyspnea over a median follow-up on 7th (*P*-value = 0.015) and 14th (*P*-value = 0.010) days after receiving the interventions which indicates a significant improvement in Levamisole/routine care group when compared to the placebo/routine care group. Individual patients’ clinical improvement in the placebo/routine care group were compared with Levamisole/ routine care and presented in detail in Table 2. The parameters of headache, asthenia, dizziness, myalgia, and nausea were also assessed on days 1, 3, 7 and 14. Our findings showed no significant difference in these variables at baseline and during the treatment (*P*-value > 0.05). As shown in Table 3, Dyspnea was evaluated based on New York Heart Association (NYHA) classification. Only one patient needed hospitalization for 14 days, which belonged to the placebo/routine care group and all the patients survived throughout or following the treatment. No adverse effects were reported by patients in either group.

**Table 2:** The Most Common Patients’ Clinical Status on Study Days 1, 3, 7, and 14

Clinical Presentation	Placebo/Routine Care	Levamisole/ Routine Care	***P*** value
	N (%)	N (%)	
**Fever**			
Day 1	20 (80.0)	24 (96.0)	0.189
Day 3	20 (80.0)	24 (96.0)	0.189
Day 7	3 (12.0)	0 (0.0)	0.235
Day 14	0 (0.0)	0 (0.0)	1.000
**Cough**			
Day 1	17 (68.0)	22 (88.0)	0.088
Day 3	17 (68.0)	23 (92.0)	0.034
Day 7	17 (68.0)	15 (60.0)	0.556
Day 14	9 (36.0)	1 (4.0)	0.005
**Dyspnea**			
Day 1	13 (52.0)	14 (56.0)	0.777
Day 3	13 (52.0)	14 (56.0)	0.777
Day 7	12 (48.0)	4 (16.0)	0.015
Day 14	0 (0.0)	7 (28.0)	0.010
**Asthenia**			
Day 1	5 (20.0)	4 (16.0)	0.490
Day 3	5 (20.0)	2 (8.0)	0.235
Day 7	4 (16.0)	1 (4.0)	0.235
Day 14	3 (12.0)	0 (0.0)	0.235
**Headache**			
Day 1	2 (8.0)	2 (8.0)	1.000
Day 3	2 (8.0)	2 (8.0)	1.000
Day 7	1 (4.0)	0 (0.0)	1.000
Day 14	1 (4.0)	0 (0.0)	1.000
**Dizziness**			
Day 1	2 (8.0)	1 (4.0)	1.000
Day 3	2 (8.0)	1 (4.0)	1.000
Day 7	2 (8.0)	0 (0.0)	0.490
Day 14	2 (8.0)	0 (0.0)	0.490
**Myalgia**			
Day 1	3 (12.0)	2 (8.0)	0.235
Day 3	3 (12.0)	2 (8.0)	0.235
Day 7	3 (12.0)	0 (0.0)	0.235
Day 14	2 (8.0)	0 (0.0)	0.490
**Nausea**			
Day 1	2 (8.0)	1 (4.0)	1.000
Day 3	2 (8.0)	1 (4.0)	1.000
Day 7	1 (4.0)	1 (4.0)	1.000
Day 14	1 (4.0)	0 (0.0)	1.000

**Table 3:** Dyspnea Evaluation Based on NYHA classification on Study Days 1 and 14

NYHA Class	Placebo/Routine Care		Levamisole/ Routine Care	
	Day 1	Day 14	Day 1	Day 14
	N (%)		N (%)	
Class I (Mild)	12	0	11	0
Class II (Mild)	0	0	0	0
Class III (Moderate)	8	4	8	0
Class IV (Severe)	5	3	6	0

Class I: No limitation of physical activity; Class II: Slight limitation of physical activity; Class III: Marked limitation of physical activity; Class IV: Unable to carry on any physical activity without discomfort [20]

As acetaminophen is the first drug of choice for fever and pain management in patients suffering from COVID-19, acetaminophen requirement was also recorded in both groups. As illustrated in Table 4, there was no significant difference between two groups in acetaminophen requirement.

**Table 4:** Acetaminophen Requirement in Patients Suffering with COVID-19

Dose of Acetaminophen	HCQ / placebo	HCQ / Levamisole	Total
	N (%)	N (%)	N (%)
**Yes**	12 (48.0)	10 (40.0)	22 (44.0)
500 mg tds for 2 days	1 (4.0)	0 (0.0)	1 (2.0)
500 mg tds for 3 days	3 (12.0)	8 (32.0)	11 (22.0)
500 mg tds for 8 days	1 (4.0)	0 (0.0)	1 (2.0)
500 mg tds for 10 days	1 (4.0)	0 (0.0)	1 (2.0)
500 mg tds for a week	5 (20.0)	2 (8.0)	7 (14.0)
500 mg tds for 2 weeks	1 (4.0)	0 (0.0)	1 (2.0)
**No**	13 (52.0)	15 (60.0)	28 (56.0)

## Discussion

The present study was a randomized controlled clinical trial evaluating the effectiveness of Levamisole in combination with routine care in patients suffering from COVID-19. The results of the current study have demonstrated that patients receiving the combination of Levamisole with routine standard of care had significantly higher chance of better clinical status including cough and dyspnea on day 14 when compared to the placebo. However, the effect-size of this finding has uncertain clinical importance. No significant difference in fever, headache, asthenia, dizziness, myalgia, and nausea was observed on days 1, 3, 7, and 14 between groups.

Currently, the coronavirus COVID-19 pandemic is a global dilemma. Since the main pathogenic mechanism of the disease is unclear, many drugs have been evaluated in an attempt to relieve the symptoms [21]. One of the suggestive agents used for systemic treatment of COVID-19 is Levamisole because of its wide variety of immunological effects including regulation of neutrophils, macrophages, and T-cell activity. Moreover, this drug also modulates the human interferon (IFNs), interleukin-6 (IL-6) and IL-8 [22, 23] and enhances the serum level of immunoglobulin A (IgA) and IgM [24]. The combination of Levamisole with ascorbic acid has shown to reverse the depressed helper/inducer subpopulation of lymphocyte in an in-vitro study. In vitro observations have further shown that the abnormality in lymphocytes could be reproduced in Levamisole-treated lymphocytes. Therefore, Levamisole could be suggested for the treatment of COVID-19 [23, 25].

As with the previous study in China [14], more than half of the infected patients in the current study were men (60%). The most common clinical presentations of 41 infected patients with COVID-19 in Wuhan, China, were the following: fever (98.0%), cough (76.0%), dyspnea (55.0%), myalgia (44.0%), sputum production (28.0%), headache (8.0%), hemoptysis (5%), and diarrhea (3%) [14]. The most common clinical presentations in the current study were fever (88.0%), cough (78.0%), and dyspnea (54.0%). While less common symptoms were asthenia (18.0%), headache (8.0%), dizziness (6.0%), myalgia (6.0%), and nausea (6.0%) at the initiation of the trial. Nevertheless, no significant difference between groups were observed.

Pro-inflammatory state is the second phase of COVID-19 disease and commonly associated with elevation in several inflammatory cytokines including IL-6 and IL-8 that results in acute lung injury, cytokine storm and systemic inflammation [15]. Thus, inflammatory response reduction could be considered as a potential therapeutic target against COVID-19 disease in this phase. Several studies are currently underway to elucidate the potential molecular mechanisms and employ some interventions to suppress this phase of COVID-19 [15, 16]. Heretofore, no specific antiviral treatment is available for COVID-19. While accurate information about the effectiveness of different lmmunomodulators are limited, anti-inflammatory and immunomodulatory therapies has the potential for COVID-19 patients in the pro-inflammatory phase [16].

In severe cases of COVID-19, the plasma level of IL-6 is especially high [26]. Using Tocilizumab (TCZ), a human IL-6 blockade agent, indicated a reduction in lung lesion opacity, oxygen demand, decrease in C-reactive protein, and normalization of lymphocytes count in 90.5, 75, 84.2, and 52.6% in COVID-19 patients, respectively [27]. Levamisole is another anti-IL-6 agent. This drug blocks pro-inflammatory activity of IL-6 and could be effective in the management of COVID-19 patients.

Although the findings of the present study are interesting, care should be taken in implementing these results and the limitations of the study should be considered. First, the main limitation was the small size of the subject groups. It should be pointed out that until now; the efficacy of Levamisole in COVID-19 management has not been evaluated. Thus, this primary evaluation is the first trial of Levamisole to gain an insight for future studies. Second, because of the urgent circumstances in which the study was done, the effect of Levamisole on SARS-CoV-2 viral load were not assessed. Third, while the dose of Levamisole was chosen based on previous data, the effective dose for COVID is yet to be determined. The last limitation is that other laboratory variables which could be used in identifying additional predictors of patients’ outcomes were not collected.

Given the small sample and the lack of power to detect an efficacy outcome difference, we cautiously interpret these data to suggest the possibility of a beneficial effect and the absence of harm. Thus, we conclude that further efficacy evaluation in the form of a larger clinical trial is warranted.

## Conclusions

Previous investigation found that lmmunomodulators play an important role in management of patients infected with COVID-19 [21, 28]. Levamisole is a safe and successful immunomodulatory drug, suggesting that it may also attenuate the exaggerated immune response associated with COVID-19 severity. Our results indicated that Levamisole can efficiently improve some of the Clinical Status when compared to placebo.

The results of the current study have demonstrated that adding Levamisole to the routine standard of care significantly enhanced the overall clinical status including cough and dyspnea in COVID-19 patients when compared to the placebo/routine care. However, the effect-size of this finding has uncertain clinical importance.

## Data Availability

All data generated or analyzed during this study are included in this published article.

## Abbreviations

SARS-CoV-2 or COVID-19: Severe Acute Respiratory Syndrome Coronavirus 2
WHO: World Health Organization
ACE-2: Angiotensin-converting enzyme 2
Th1: CD_4_^+^-T-helper
LPV/r: Lopinavir/ritonavir
NSAIDs: Nonsteroidal anti-inflammatory drugs
CQ: Chloroquine
HCQ: Hydroxychloroquine
IL-6: Interleukin-6
IFNs: Human interferon
IgA: Immunoglobulin A
IgM: Immunoglobulin M
CONSORT: Consolidated Standards of Reporting Trials
NYHA: New York Heart Association
IL: Interleukins
TCZ: Tocilizumab
